# ^68^Ga-DOTA-DiPSMA PET/CT Imaging: Biodistribution, Dosimetry, and Preliminary Application in Prostate Cancer

**DOI:** 10.3389/fbioe.2021.811972

**Published:** 2022-01-28

**Authors:** Jiaying Zhang, Zefang Lin, Xiaojun Zhang, Rong Lin, Mengchao Cui, Weibing Miao, Shaobo Yao

**Affiliations:** ^1^ Department of Nuclear Medicine, The First Affiliated Hospital of Fujian Medical University, Fuzhou, China; ^2^ Key Laboratory of Radiopharmaceuticals, Ministry of Education, Beijing Normal University, Beijing, China; ^3^ Fujian Provincial Key Laboratory of Precision Medicine for Cancer, The First Affiliated Hospital of Fujian Medical University, Fuzhou, China

**Keywords:** prostate cancer, ^68^Ga-DOTA-DiPSMA, biodistribution, dosimetry, PET/CT

## Abstract

**Purpose**: This prospective trial aimed to evaluate the safety, dosimetry, and biodistribution of a novel theranostic probe ^68^Ga-DOTA-DiPSMA. Also, we have performed the first preliminary application with ^68^Ga-DOTA-DiPSMA in prostate cancer (PCa) patients.

**Methods**: Five healthy volunteers and ten PCa patients were injected with an intravenous bolus of ^68^Ga-DOTA-DiPSMA. They received serial whole-body PET scans from the time of injection up to 60 min post-injection, with a second PET/CT scanning at 120 min post-injection. In PCa patients, low-dose CT scan and whole-body PET were performed with 2 min per bed position in 40 min post-injection. Absorbed organ doses and effective doses were calculated using OLINDA/EXM. Normal organ uptake and tumor lesion uptake were measured. A lesion-by-lesion analysis was performed.

**Results**: ^68^Ga-DOTA-DiPSMA administration was safe and well-tolerated. The kidneys received the highest absorbed dose (114.46 ± 29.28 μSv/MBq), followed by the urinary bladder wall (100.82 ± 46.22 μSv/MBq) in accordance with the expected Prostate-Specific Membrane Antigen (PSMA) renal excretion of the tracer. The mean effective dose was 19.46 ± 1.73 μSv/MBq. The SUV_max_ of ^68^Ga-DOTA-DiPSMA PET/CT for PCa lesions, bone metastases, and lymph node metastases was 4.41 ± 2.72, 2.95 ± 1.11, and 3.26 ± 1.20, respectively.

**Conclusion**: Injection of ^68^Ga-DOTA-DiPSMA is safe and associated with low absorbed and effective doses. ^68^Ga-DOTA-DiPSMA shows favorable kinetics and imaging characteristics in patients who warrant further head-to-head comparison to validate ^68^Ga-DOTA-DiPSMA as an alternative for gallium-68-labeled PSMA clinical PET. Low nonspecific uptake in normal organs of ^68^Ga-DOTA-DiPSMA indicates potential radioligand therapy (RLT) application when labeled with ^177^Lu, ^90^Y, or ^225^Ac.

## Introduction

Prostate cancer (PCa) is one of the most frequently diagnosed cancers in men and the lethal malignant diseases leading to male cancer-related death worldwide (Attard et al., 2016). The accurate presence and location of primary or recurrent tumors are critical for planning effective patient management (Mottet et al., 2021).

The diagnostic capability of conventional anatomic imaging such as MRI and CT to determine PCa is limited in metastases and specificity (Vos et al., 2013). Only prostate biopsy is the definitive way to confirm PCa (Attard et al., 2016). Multiple needle biopsies will increase the positive rate of lesion determination significantly. However, it is difficult to determine distant metastases and increase the risk of complications resulting from biopsy operation (Attard et al., 2016). There has been an unmet need for more advanced imaging modalities to determine primary and metastatic lesions that can be helpful to PCa patient management (observation, salvage local therapy, and systemic therapy). PET with ^18^F-FDG is effective for most malignant tumors, but it lacks sensitivity for PCa. Therefore, it is urgent to discover new nuclear medicine imaging agents with more specificity for PCa.

Prostate-Specific Membrane Antigen (PSMA) is a transmembrane glycoprotein enzyme selectively overexpressed in PCa cells, with its expression increasing in higher-grade malignancy (Bouchelouche et al., 2010). PET imaging with PSMA probes targeting various PCa-specific markers will provide additional molecular information to facilitate lesion detection and staging (Perera et al., 2020).

Recently, a relatively new nuclear imaging modality ^68^Ga-PSMA PET/CT imaging with good PCa diagnosis and staging performance has become increasingly utilized to evaluate PCa aggressiveness, especially in patients with biochemical recurrence after surgery (Sachpekidis et al., 2016; Koerber et al., 2017; Wang et al., 2020). PSMA can be coupled with different chelators and labeled with corresponding radionuclides for different purposes. The most widely used PSMA ligands in the clinical examination are PSMA-11 and PSMA-617 containing different linkers and chelators. According to the previously published papers, ^68^Ga-PSMA-11 and ^177^Lu-PSMA-617 are a molecular pair in metastatic castration-resistant PCa (mCRPC) diagnosis and radioligand therapy (RLT) (Rahbar et al., 2017; Sun et al., 2020; Violet et al., 2020). However, considering the high nonspecific uptake in the salivary, kidneys, and bone marrow of ^68^Ga-PSMA-11 and ^177^Lu-PSMA-617, novel PSMA tracers with lower accumulation in normal organs are urgently needed.

**FIGURE 1:**
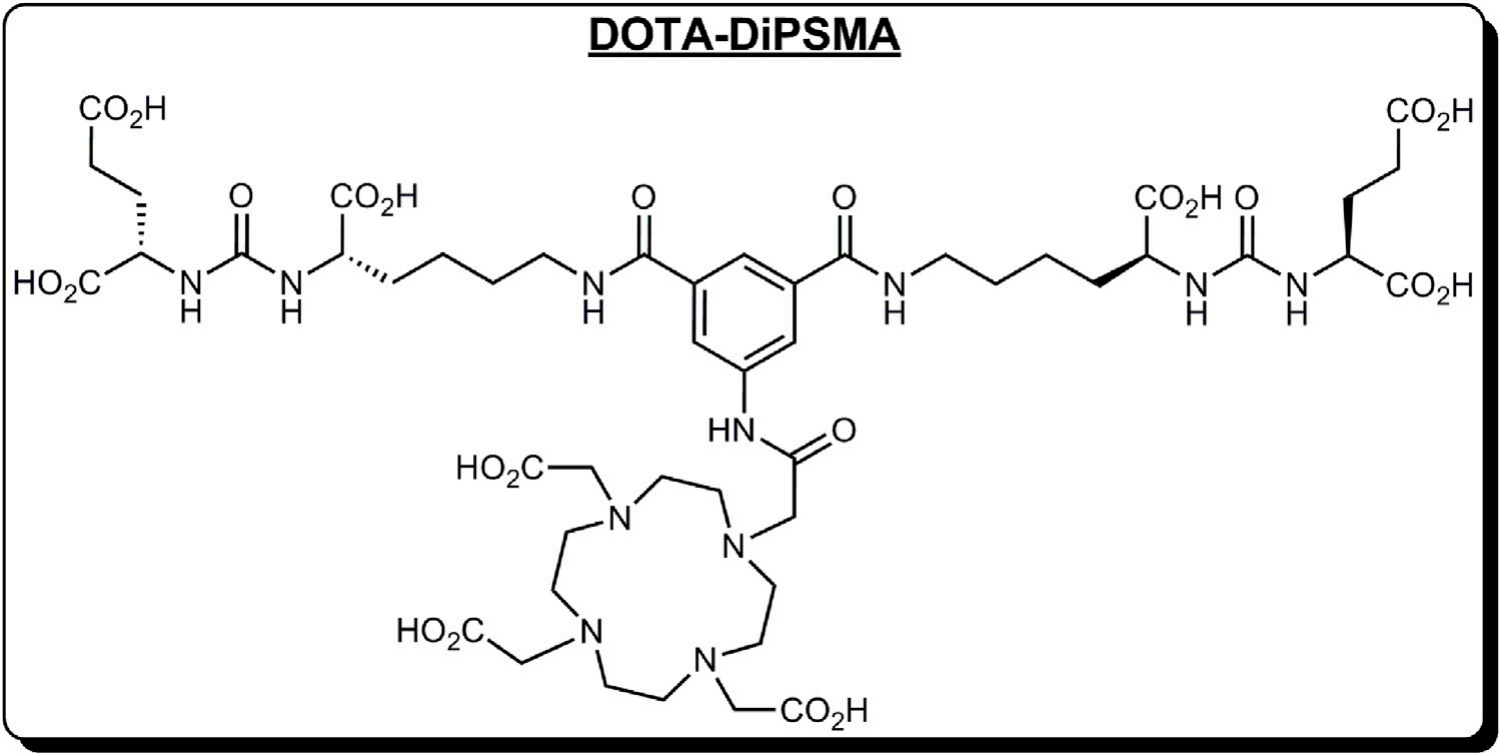
Structure of DOTA-DiPSMA.

We have discovered a new PSMA dimer (DOTA-DiPSMA, Figure 1). The Prof. Cui group from Beijing Normal University will discuss the discovery and preclinical experiments, which will be published elsewhere. Preclinical experiments proved its good imaging ability and low unspecific uptake in normal organs including the liver, kidneys, spleen, and salivary glands. In addition, the affinity of DiPSMA-DOTA-COOH to the PSMA receptor can reach 1.56 nM. This was the first study in humans, following the abovementioned preclinical studies. In this study, we aimed to evaluate the safety, biodistribution, and dosimetry of ^68^Ga-DOTA-DiPSMA in healthy volunteers and its diagnostic efficacy in PCa patients.

## Methods

### Healthy Volunteers and Patients

This study was approved by the Independent Ethics Committee of First Affiliated Hospital of Fujian Medical University [No. MRCTA, ECFAH of FMU (2019)293]. All subjects gave written informed consent and were registered at ClinicalTrials.gov (NCT04525612). Five healthy volunteers and ten patients were enrolled in this study. Five healthy volunteers [5 men, age range 42–76 years (mean age ± SD, 59.83 ± 11.65 years); weight range, 55.0–78.0 kg (mean weight ± SD, 70.27 ± 13.05 kg)] were enrolled to validate the safety, biodistribution, and radiation dosimetry of ^68^Ga-DOTA-DiPSMA in this study. Exclusion criteria consisted of mental illness conditions, severe liver or kidney disease with serum creatinine greater than 3.0 mg/dl, or any hepatic enzyme level 5 times or more than the standard upper limit. Participants were also excluded if they were known to have severe allergy or hypersensitivity to intravenous radiographic contrast or claustrophobia during PET/CT scanning.

A total of 10 patients who were newly diagnosed as having PCa by sextant core-needle biopsy and had not received any prior therapy were enrolled with written informed consent. The inclusion criteria were those aged between 40 and 80 years, who have a prostate neoplasm identified by ultrasound or MRI, and were diagnosed by needle biopsy as having PCa. The exclusion criteria included claustrophobia, kidney or liver failure, and inability to fulfill the study. The demographics of healthy volunteers and patients are listed in Table 1.

**TABLE 1:** Summary of the healthy volunteer and patient characteristics and PET findings in ten newly diagnosed prostate cancer patients with ^68^Ga-DOTA-DiPSMA.

No.	Age (year)	Sex	Weight (kg)	Inject dose	Serum PSA (ng/ml)	SUV_max_ for detected lesions	Lesion location
HV1	55	M	73.8	3.3	—	—	—
HV2	42	M	92.3	5.5	—	—	—
HV3	67	M	60.2	3.8	—	—	—
HV4	56	M	74.3	5.44	—	—	—
HV5	50	M	65.9	5.5	—	—	—
1	68	M	51.8	3.6	26.95	3.3	Prostate
						1.9	T8 vertebrae
						4.1	Sacrum
						3.7	Iliac
						2.1	Iliac
2	76	M	67.2	3.0	10.5	2.9	Prostate
						2.6	Prostate
3	67	M	61	4.1	10.3	1.9	Prostate
						1.7	Prostate
						3.7	Prostate
4	71	M	92.4	3.05	9.56	6.4	Prostate
						3.8	Prostate
5	73	M	67	3.06	8.62	4.0	Prostate
6	77	M	65.1	3.85	6.27	2.1	Prostate
						2.3	Prostate
7	77	M	57.9	2.70	17.22	9.76	Prostate
8	69	M	65.6	2.98	37.9	10.6	Prostate
						3.0	Prostate
						5.8	Seminal vesicle
						2.1	Seminal vesicle
9	78	M	56	2.8	35.72	6.6	Prostate
10	80	M	68	2.8	60.1	5.9	Prostate
						2.6	Iliac lymph node
						3.1	Iliac lymph node
						4.6	Iliac lymph node
						4.3	Iliac lymph node
						1.7	Iliac lymph node

Note. PSA, prostate-specific antigen.

Subject age averaged 73.6 ± 4.6 years (median 74.5 years; range 67–80 years), and body mass averaged 65.2 ± 11.0 kg (median 65.4 kg; range 51.8–92.4 kg). Serum prostate-specific antigen (PSA) values averaged 22.3 ± 17.6 ng/ml (median 13.9 ng/ml; range 6.27–60.1 ng/ml). The reported serum PSA levels were the most recent clinical values at the time of PET/CT imaging. Subjects were numbered chronologically in the order of imaging with ^68^Ga-DOTA-DiPSMA.

### Safety Assessment

Patient safety was assessed and graded according to Common Terminology Criteria for Adverse Events (version 5.0), electrocardiograms, physical examination, and vital signs (blood pressure, respiratory rate, heart rate, and body temperature). Within the first 72 h after ^68^Ga-DOTA-DiPSMA injection, the research team kept phone contact with each subject, monitoring their adverse event (AE) responses.

### Radiopharmaceutical Preparation

Precursors were supplied by Prof. Cui from Key Laboratory of Radiopharmaceuticals, Ministry of Education, Beijing Normal University. Radiolabeling of DiPSMA-DOTA-COOH was performed in a sterile hot cell manually. ^68^Ga^3+^ was eluted from a ^68^Ge/^68^Ga generator (JSC Isotope, Moscow, Russia) using 0.1 M of HCl. The clinical doses of DOTA-DiPSMA (30 μg) were compounded in 1.25 M of NaOAc buffer to adjust pH to around 4.0 and labeled with an average of 585.34 ± 177.97 MBq (15.82 ± 4.81 mCi) of ^68^Ga^3+^ using a reaction temperature of 95°C for 10 min. Our protocol permits the radiochemical purity of the product ^68^Ga-DOTA-DiPSMA to exceed 99% so that we omit the purification step. The final product will pass through a sterile filter membrane (Millipore, Billerica, MA, United States) and then be diluted to 5 ml in a sterile syringe for injection. The total time required for completion of radiolabeling and quality control averaged approximately 30 min. Quality control items are shown in Table 2.

**TABLE 2:** Quality control test items.

Test item	Acceptance criteria	Test method
Appearance	Colorless and particle-free	Visual inspection
pH	3.5–6.0	pH strip
Radiochemical purity	—	Radio TLC
^68^Ga-DOTA-DiPSMA	≥90%	—
Maximum injection volume	≤5 ml	Injector
Sterility	No growth after 14 days of incubation at 37°C	Petri dish inoculation method
Bacterial endotoxins	≤15 EU per ml	LAL test

Note. TLC, thin-layer chromatography; LAL, limulus amebocyte lysate.

### Examination Procedures

For healthy volunteers, the blood pressure, pulse, respiratory frequency, and temperature were measured; and routine blood and urine tests, liver function, and renal function were examined immediately before and 24 h after the scan. In addition, any possible side effects during ^68^Ga-DOTA-DiPSMA PET/CT scanning and within 1 week after the examination were collected and analyzed. No specific subject preparation was requested on the day of ^68^Ga-DOTA-DiPSMA PET/CT. For the volunteers, after the whole-body low-dose CT scan, 111–222 MBq (3–6 mCi) of ^68^Ga-DOTA-DiPSMA were injected intravenously, followed by serial whole-body PET acquisitions. The whole body (from the top of the skull to the middle of the femur) of each volunteer was covered by 6 bed positions. The acquisition duration was 2 min/bed position at 5, 15, 30, 45, 60, and 120 min after injection.

For the patients, ^68^Ga-DOTA-DiPSMA PET/CT scanning was performed at 40 min after tracer administration. For each patient, 103.6–151.7 MBq (2.8–4.1 mCi) of ^68^Ga-DOTA-DiPSMA was injected intravenously. After a low-dose CT scan, whole-body PET was performed with 2 min per bed position (5–6 bed positions depending on the patient’s height). The emission data were corrected for randomness, dead time, scattering, and attenuation. The conventional reconstruction algorithm was used, and the images were zoomed with a factor of 1.2. The images were transferred to an MMWP workstation (Siemens, Erlangen, Germany) for analysis.

### Biodistribution Assessment and Dosimetry

Image analysis was performed using MIM v6.9.4 (MIM Software Inc., Cleveland, OH, United Ststes). The volume of interests (VOIs) were drawn over healthy organs on all ^68^Ga-DOTA-DiPSMA PET images, and SUV_mean_ in these VOIs was determined to obtain the biodistribution of this tracer. Tumor lesions were evaluated in consensus by two nuclear medicine physicians.

All source organs with relevant detectable activity were delineated on the PET images with CT guidance for the healthy volunteers, using MIM software v6.9.4. Time-integrated activity coefficients (normalized cumulated activity (NCA)) were calculated for each source organ by integrating their time–activity curves through curve fitting and normalizing the cumulated activity to the injected activity. Based on the time-integrated activity coefficients, individual absorbed organ doses and the effective dose were determined using OLINDA/EXM v1.1 (Vanderbilt University, Nashville, TN, United States). Calculations were performed with modeling of urinary bladder voiding. Parameters representing the fraction leaving the body *via* urine and biologic half-time were obtained from the fit and used to model urinary bladder voiding. Urinary bladder voiding models with voiding intervals of 1 h were applied. The 70-kg adult male models were used. Organ-absorbed doses, effective doses, and effective dose equivalents were calculated as mean ± SD across subjects. SPSS 23.0 Software (IBM SPSS, Chicago, IL, United States) was used for statistical analyses.

## Result

### Patient Safety

^68^Ga-DOTA-DiPSMA was found to be safe and well-tolerated in all subjects. No AEs or serious AEs occurred after ^68^Ga-DOTA-DiPSMA injection for all the healthy volunteers and patients. No apparent changes in vital signs or clinical laboratory tests were found before and after the injection of ^68^Ga-DOTA-DiPSMA.

### Biodistribution

**FIGURE 2:**
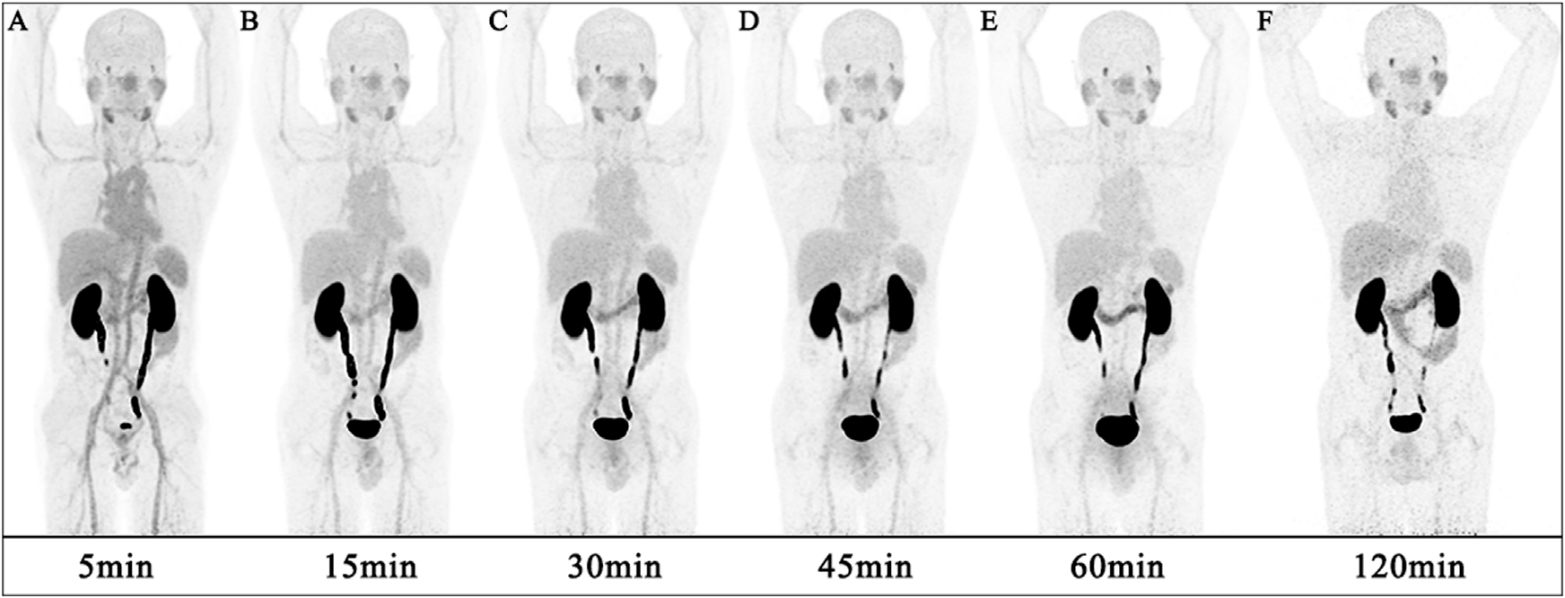
A maximum intensity projection PET images at several time points post-injection of a 56-year-old male healthy volunteer.

**FIGURE 3:**
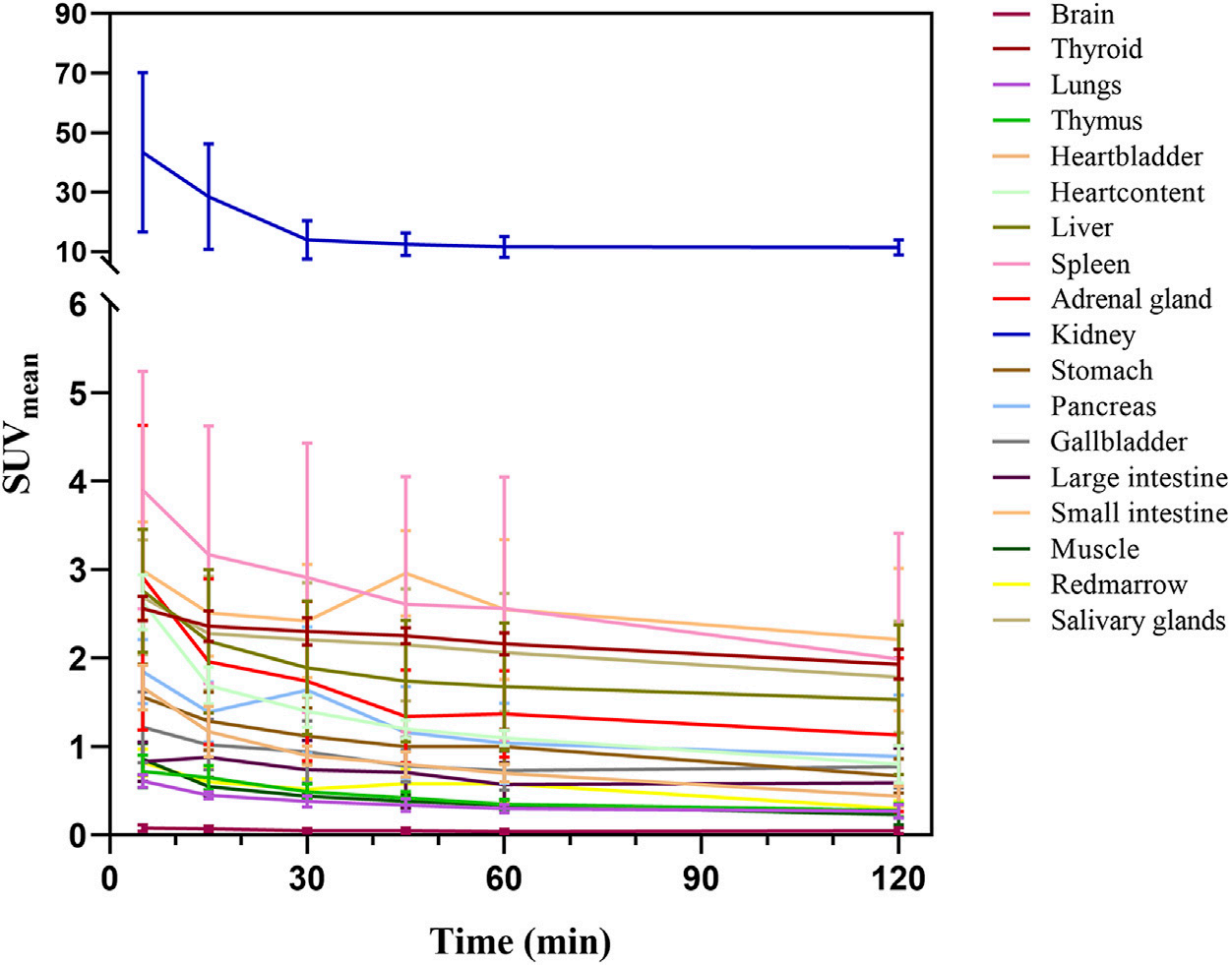
Biodistribution of ^68^Ga-DOTA-DiPSMA in healthy volunteers.

Figures 2, 3 illustrate the biodistribution of ^68^Ga-DOTA-DiPSMA as a function of time in healthy volunteers. The whole-body background of ^68^Ga-DOTA-DiPSMA was low. The highest uptake was observed in the kidney with a SUV_mean_ of 43.4 ± 26.8 at 5 min p.i. and further decreased to 11.4 ± 6.5 at 120 min p.i. The spleen, liver, salivary gland, and small intestine showed moderate uptake, with SUV_mean_ of 2.90 ± 1.5, 1.89 ± 0.75, 2.30 ± 0.87, and 2.42 ± 0.64 at 30 min after injection, respectively. Low background uptakes were observed in the brain, lungs, muscle, red marrow, heart, thyroid, gall bladder, pancreas, stomach, bone, and large intestine. The rapid presence in the kidneys, followed by a passage toward the urinary bladder, illustrated the tracer’s fast and mainly renal excretion.

### Dosimetry

The average estimated absorbed organ in healthy volunteers is summarized in Table 3. The highest absorbed dose was received by the kidneys (114.46 ± 29.28 μSv/MBq), followed by the urinary bladder wall (100.82 ± 46.22 μSv/MBq). The mean effective dose was 19.46 ± 1.73 μSv/MBq.

**TABLE 3:** Estimated absorbed organ doses and effective dose for ^68^Ga-DOTA-DiPSMA in healthy volunteers.

Absorbed dose	Mean (μSv/MBq)	SD (μSv/MBq)
Adrenals	14.44	0.34
Brain	11.16	0.43
Breasts	10.94	0.42
Gallbladder wall	13.12	3.33
LLI wall	12.47	3.71
Small intestine	12.36	3.61
Stomach wall	11.74	3.51
ULI wall	12.18	3.57
Heart wall	11.38	3.48
Kidneys	114.46	29.28
Liver	24.76	5.38
Lungs	12.12	0.41
Muscle	12.24	0.30
Ovaries	14.26	0.13
Pancreas	14.56	0.31
Red marrow	11.38	3.74
Osteogenic cells	14.53	5.46
Skin	10.68	0.33
Spleen	30.98	7.76
Testes	12.38	0.13
Thymus	12.14	0.48
Thyroid	12.04	0.48
Urinary bladder wall	100.82	46.22
Uterus	15.54	0.61
Salivary glands	24.32	7.11
Total body	9.71	4.93
Effective dose equivalent	26.42	3.68
Effective dose	19.46	1.73

Note. LLI, lower large intestine; ULI, upper large intestine.

### Detection of Primary Prostate Cancer

**FIGURE 4:**
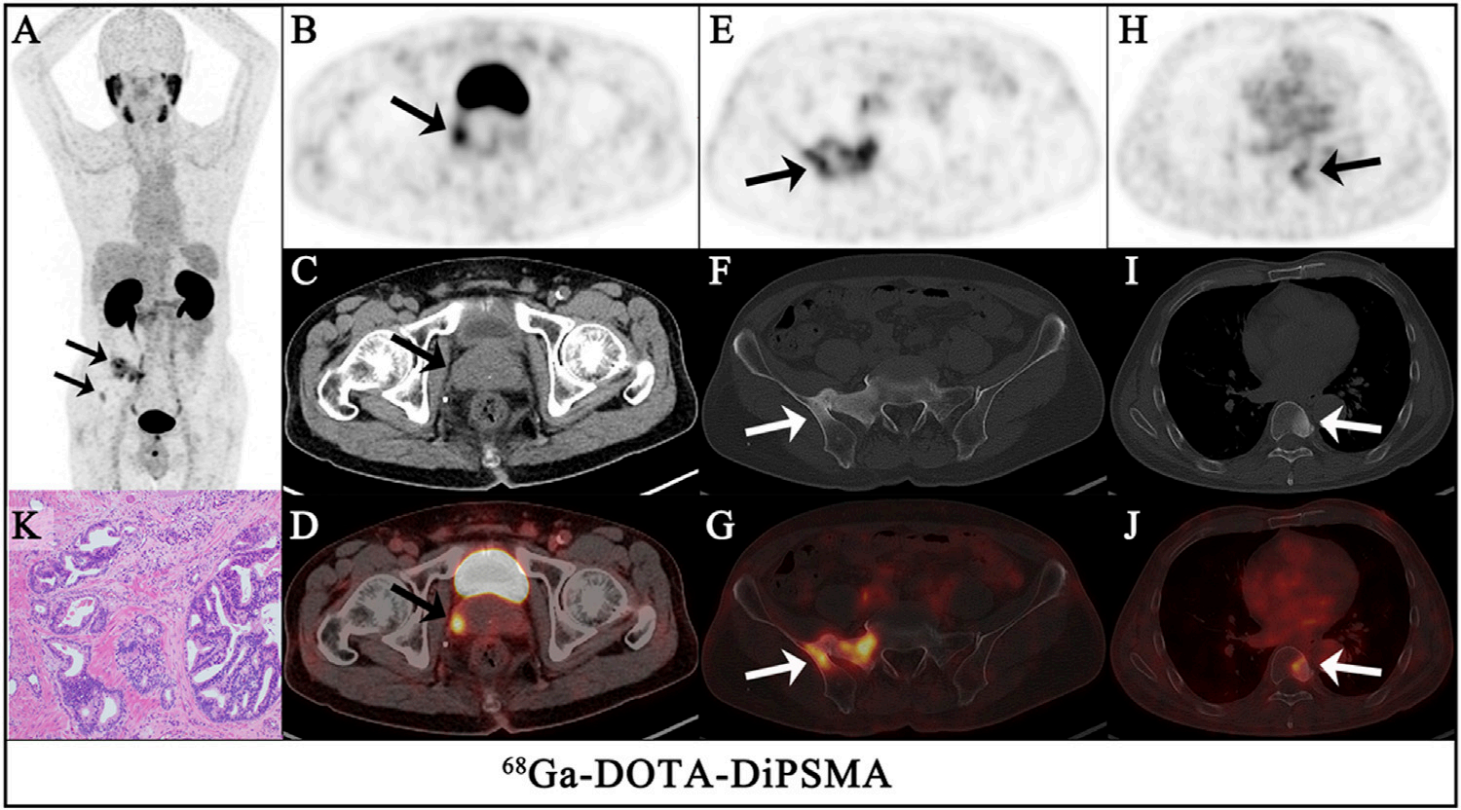
A 68-year-old-man had confirmed prostate cancer after a needle biopsy of the prostate for 1 week. The maximum intensity projection (MIP) of ^68^Ga-DOTA-DiPSMA PET/CT [**(A)**, arrows] showed significantly abnormal uptake in the image. Axial views of the prostate (top, PET; middle, CT; bottom, fusion image) show intense uptake (SUV_max_ 3.3) in the isodense nodule of the prostate [**(B–D)**, arrows]. In the other level axial views, increased ^68^Ga-DOTA-DiPSMA uptake was observed in the sacrum and iliac [**(E–G)**, arrows] and the 8th thoracic vertebrae lesions [**(H–J)**, arrows], which were concomitant with bone density increased. Postoperative pathology confirmed it as adenocarcinoma of the prostate (K).

**FIGURE 5:**
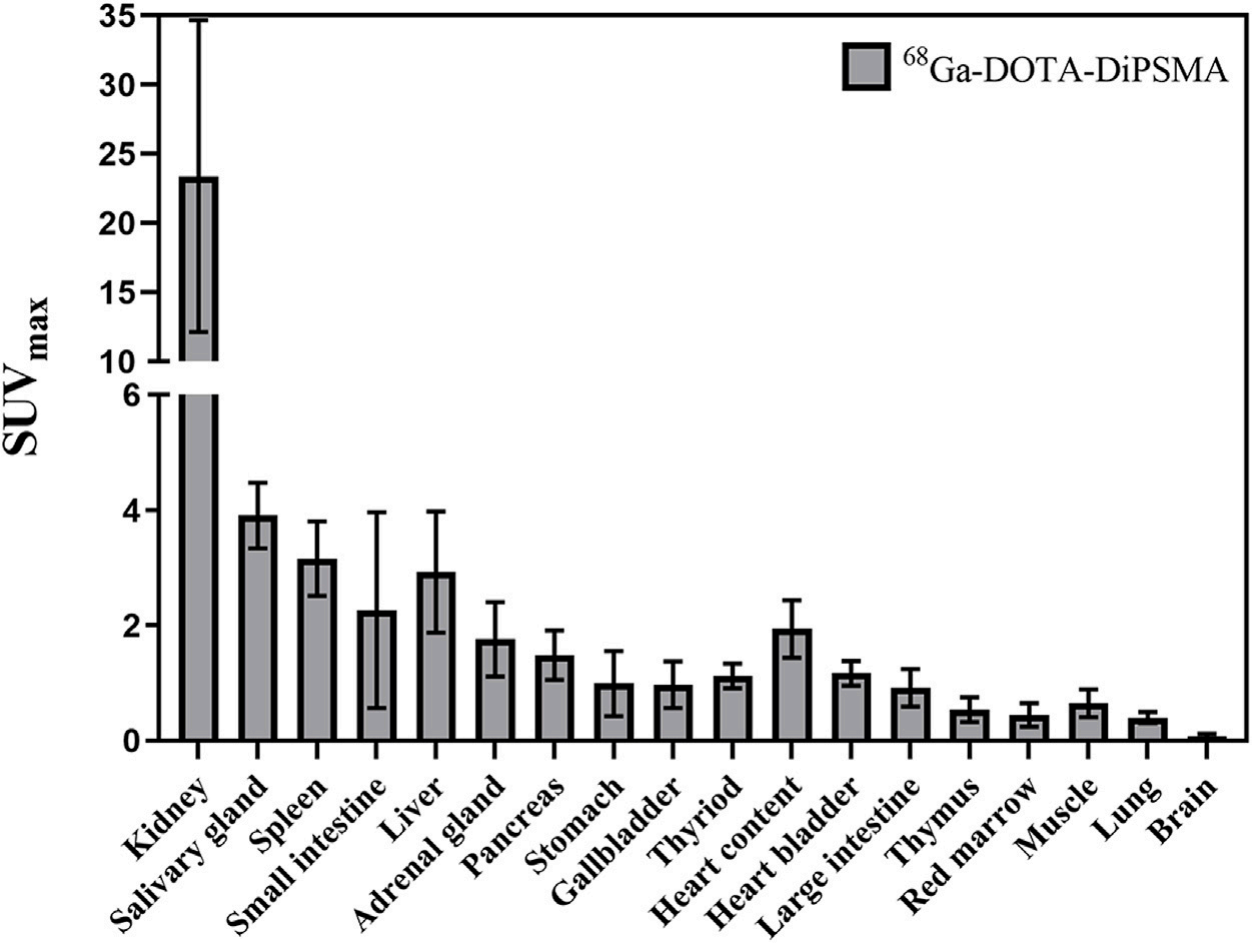
Normal organs’ SUV_max_ (average in 10 patients) of ^68^Ga-DOTA-DiPSMA.

For the 10 patients with primary PCa, ^68^Ga-DOTA-DiPSMA PET/CT showed 27 positive findings, including 16 prostate lesions, 4 bone metastases, 5 lymph node metastases, and 2 seminal vesicle metastases. The primary lesions were confirmed by needle biopsy. SUV_max_ for prostate lesions, bone metastases, and lymph node metastases were 4.41 ± 2.72, 2.95 ± 1.11, and 3.26 ± 1.20, respectively (Table 1; Figure 4). Low background uptake was observed in ^68^Ga-DOTA-DiPSMA (the salivary glands SUV_max_ 4.88 ± 2.04, liver SUV_max_ 2.92 ± 1.05, kidneys SUV_max_ 23.40 ± 11.27, and spleen SUV_max_ 3.16 ± 0.64) (Table 4; Figure 5).

**TABLE 4:** Different organs’ SUV_max_ for ^68^Ga-DOTA-DiPSMA in PCa patients.

	SUV_max_	SD
Thyriod	1.12	0.21
Brain	0.07	0.04
Lung	0.40	0.09
Thymus	0.53	0.19
Heart bladder	1.18	0.21
Heart content	1.94	0.50
Liver	2.92	1.05
Kidney	23.40	11.27
Stomach	0.99	0.57
Adrenal gland	1.76	0.64
Pancreas	1.48	0.43
Spleen	3.16	0.64
Gallbladder	0.97	0.41
Large intestine	0.92	0.33
Small intestine	2.26	1.70
Red marrow	0.44	0.20
Muscle	0.65	0.24
Salivary gland	4.88	2.04
Prostate lesions	4.41	2.72
Lymph node metastasis	3.26	1.20
Bone metastasis	2.95	1.11
Seminal vesicle metastasis	3.95	2.62

Note. PCa, prostate cancer.

## Discussion

To our knowledge, this was the first human study to evaluate the novel tracer ^68^Ga-DOTA-DiPSMA in healthy volunteers and patients with PCa. This tracer is a new type of ^68^Ga-labeled dimer PSMA imaging agent with a simple structure, easy synthesis, and low synthesis cost. ^68^Ga-DOTA-DiPSMA can be prepared by a one-step labeling reaction in a high yield greater than 95% between ^68^Ga^3+^ ions eluted from a germanium–gallium generator and the precursor DiPSMA-DOTA-COOH.

Here, we presented the results of an independently performed first clinical evaluation of ^68^Ga-DOTA-DiPSMA in five healthy volunteers, including biodistribution, dosimetry, and safety. Also, we have performed the first initial application with ^68^Ga-DOTA-DiPSMA in PCa patients. The results showed that this tracer displayed favorable biodistribution and dosimetry features and was well-tolerated in all patients. ^68^Ga-DOTA-DiPSMA showed high PSMA affinity. The biodistribution of ^68^Ga-DOTA-DiPSMA was similar to that of ^68^Ga-PSMA-11 (Pfob et al., 2016). The rapid presence in the kidneys, followed by a passage toward the urinary bladder, illustrates the tracer’s fast and mainly renal excretion. The highest uptake was observed in the kidneys and rapidly cleared through the urinary system in both tracers, consistent with the published ^68^Ga-PSMA-11 results (Afshar-Oromieh et al., 2016; Zamboglou et al., 2016; Chen et al., 2019; Sandgren et al., 2019). However, ^68^Ga-DOTA-DiPSMA observed SUV_mean_ values at 60 min for the kidneys, liver, spleen, and parotids (11.93 ± 3.54, 1.68 ± 0.72, 2.56 ± 1.49, and 2.16 ± 0.89, respectively) were in general lower than ^68^Ga-PSMA-11 (30.1 ± 6.6, 3.3 ± 0.6, 5.2 ± 2.5, and 9.4 ± 2.0, respectively) (Green et al., 2017).

The dosimetry data of ^68^Ga-DOTA-DiPSMA showed a little lower yet comparable effective dose than ^68^Ga-PSMA-11 (0.019 vs. 0.022/0.023 mSv/MBq) (Afshar-Oromieh et al., 2016; Sandgren et al., 2019), salivary glands (0.024 vs. 0.089 mSv/MBq), kidney (0.114 vs. 0.240 mSv/MBq), liver (0.0240 vs. 0.053 mSv/MBq), and spleen (0.031 vs. 0.046 mSv/MBq) (Sandgren et al., 2019). We thought that the lower liver and spleen dose of ^68^Ga-DOTA-DiPSMA might be attributed to the dosimetry methodology.

It is crucial to reduce the radiation dose of nonspecific organs and tissues in the field of radionuclide therapy (RLT) (ICRP, 2002). ^177^Lu-PSMA-617 RLT is a promising option for patients with mCRPC (Delker et al., 2016; Kratochwil et al., 2016; Paganelli et al., 2020; Rasul et al., 2020). Based on the lower nonspecific uptake and effective dose of ^68^Ga-DOTA-DiPSMA, the radiation dosimetry in normal organs seemed to be reduced when DOTA-DiPSMA was labeled with ^177^Lu for RLT. Low background uptakes were observed in the brain, lungs, muscle, red marrow, heart, thyroid, gall bladder, pancreas, stomach, bone, and large intestine. The low uptake of the dimer DOTA-DiPSMA in the parotid glands and the clearance in the kidneys were impressive, which could be an advantage for RLT.

A critical finding of our study is the high tumor accumulation of ^68^Ga-DOTA-DiPSMA, which showed high tumor uptakes with the highest SUV_max_ up to 10.6 on ^68^Ga-DOTA-DiPSMA. The primary lesions showed the highest uptake (SUV_max_ 4.41 ± 2.72). For metastasis lesions, the highest uptake was shown in a seminal vesicle (SUV_max_ 3.95 ± 2.61), followed by the iliac lymph node (SUV_max_ 3.26 ± 1.20), and the lowest uptake was observed in the bone (SUV_max_ 2.95 ± 1.11). However, the lower uptake of ^68^Ga-DOTA-DiPSMA in normal organs may be its advantage (Afshar-Oromieh et al., 2015; Fendler et al., 2017). The study on ^68^Ga-DOTA-DiPSMA provided a new radiotracer targeting PSMA to diagnose PCa. It was conducive to the accuracy of PCa staging. The small lesion near the urinary bladder would be more apparent with this relatively low background. The mechanism of DOTA-DiPSMA in reducing the uptake in the salivary gland and kidney was still unknown, which needs further studies to confirm.

The primary limitation of our study is the sample size, which did not enable accurate multivariate regression analysis in comparing the diagnosis efficacy of ^68^Ga-DOTA-DiPSMA with ^68^Ga-PSMA-11, which is the next work in our research group. Besides, neither blood nor urine samples were collected in our study, which will allow for the stability test *in vivo*. Further detailed and head-to-head comparison studies are required.

## Conclusion

^68^Ga-DOTA-DiPSMA is safe and well-tolerated and shows favorable dosimetry and biodistribution in healthy volunteers and detection performances in PCa patients. The low uptake of the dimer DOTA-DiPSMA in the parotid glands and the clearance in the kidneys were impressive. The lower background of ^68^Ga-DOTA-DiPSMA showed its potential application for RLT when labeled with ^177^Lu. DOTA-DiPSMA is a promising novel theranostic tracer for both PCa patient diagnosis and RLT. Further validation by head-to-head comparison is warranted.

## Data Availability

The original contributions presented in the study are included in the article/supplementary material, further inquiries can be directed to the corresponding author.

## References

[B1] Afshar-Oromieh A. Avtzi E. Giesel F. L. Holland-Letz T. Linhart H. G. Eder M. (2015). The Diagnostic Value of PET/CT Imaging with the 68Ga-labelled PSMA Ligand HBED-CC in the Diagnosis of Recurrent Prostate Cancer. Eur. J. Nucl. Med. Mol. Imaging. 42, 197–209. 10.1007/s00259-014-2949-6 25411132 PMC4315487

[B2] Afshar-Oromieh A. Hetzheim H. Kübler W. Kratochwil C. Giesel F. L. Hope T. A. (2016). Radiation Dosimetry of 68Ga-PSMA-11 (HBED-CC) and Preliminary Evaluation of Optimal Imaging Timing. Eur. J. Nucl. Med. Mol. Imaging. 43, 1611–1620. 10.1007/s00259-016-3419-0 27260521

[B3] Attard G. Parker C. Eeles R. A. Schröder F. Tomlins S. A. Tannock I. (2016). Prostate Cancer. The Lancet. 387, 70–82. 10.1016/S0140-6736(14)61947-4 26074382

[B4] Bouchelouche K. Choyke P. L. Capala J. (2010). Prostate Specific Membrane Antigen- a Target for Imaging and Therapy with Radionuclides. Discov. Med. 9, 55–61. PMID: 20102687. 20102687 PMC3410553

[B5] Chen M. Zhang Q. Zhang C. Zhao X. Marra G. Gao J. (2019). Combination of 68Ga-PSMA PET/CT and Multiparametric MRI Improves the Detection of Clinically Significant Prostate Cancer: A Lesion-By-Lesion Analysis. J. Nucl. Med. 60, 944–949. 10.2967/jnumed.118.221010 30552201 PMC6604689

[B6] Delker A. Fendler W. P. Kratochwil C. Brunegraf A. Gosewisch A. Gildehaus F. J. (2016). Dosimetry for 177Lu-DKFZ-PSMA-617: a New Radiopharmaceutical for the Treatment of Metastatic Prostate Cancer. Eur. J. Nucl. Med. Mol. Imaging. 43, 42–51. 10.1007/s00259-015-3174-7 26318602

[B7] Fendler W. P. Eiber M. Beheshti M. Bomanji J. Ceci F. Cho S. (2017). 68Ga-PSMA PET/CT: Joint EANM and SNMMI Procedure Guideline for Prostate Cancer Imaging: Version 1.0. Eur. J. Nucl. Med. Mol. Imaging. 44, 1014–1024. 10.1007/s00259-017-3670-z 28283702

[B8] Green M. A. Eitel J. A. Fletcher J. W. Mathias C. J. Tann M. A. Gardner T. (2017). Estimation of Radiation Dosimetry for 68Ga-HBED-CC (PSMA-11) in Patients with Suspected Recurrence of Prostate Cancer. Nucl. Med. Biol. 46, 32–35. 10.1016/j.nucmedbio.2016.11.002 28012435

[B9] ICRP (2002). Basic Anatomical and Physiological Data for Use in Radiological protection: Reference Values. A Report of Age- and Gender-Related Differences in the Anatomical and Physiological Characteristics of Reference Individuals. ICRP Publication 89. Ann. ICRP. 8932, 5–265. PMID: 14506981. 10.3109/14653249.2010.487901 14506981

[B10] Koerber S. A. Utzinger M. T. Kratochwil C. Kesch C. Haefner M. F. Katayama S. (2017). 68Ga-PSMA-11 PET/CT in Newly Diagnosed Carcinoma of the Prostate: Correlation of Intraprostatic PSMA Uptake with Several Clinical Parameters. J. Nucl. Med. 58, 1943–1948. 10.2967/jnumed.117.190314 28619734

[B11] Kratochwil C. Giesel F. L. Stefanova M. Benešová M. Bronzel M. Afshar-Oromieh A. (2016). PSMA-Targeted Radionuclide Therapy of Metastatic Castration-Resistant Prostate Cancer with 177Lu-Labeled PSMA-617. J. Nucl. Med. 57, 1170–1176. 10.2967/jnumed.115.171397 26985056

[B12] Mottet N. van den Bergh R. C. N. Briers E. Van den Broeck T. Cumberbatch M. G. De Santis M. (2021). EAU-EANM-ESTRO-ESUR-SIOG Guidelines on Prostate Cancer-2020 Update. Part 1: Screening, Diagnosis, and Local Treatment with Curative Intent. Eur. Urol. 79, 243–262. 10.1016/j.eururo.2020.09.042 33172724

[B13] Paganelli G. Sarnelli A. Severi S. Sansovini M. Belli M. L. Monti M. (2020). Dosimetry and Safety of 177Lu PSMA-617 Along with Polyglutamate Parotid Gland Protector: Preliminary Results in Metastatic Castration-Resistant Prostate Cancer Patients. Eur. J. Nucl. Med. Mol. Imaging. 47, 3008–3017. 10.1007/s00259-020-04856-1 32430583

[B14] Perera M. Papa N. Roberts M. Williams M. Udovicich C. Vela I. (2020). Gallium-68 Prostate-specific Membrane Antigen Positron Emission Tomography in Advanced Prostate Cancer-Updated Diagnostic Utility, Sensitivity, Specificity, and Distribution of Prostate-Specific Membrane Antigen-Avid Lesions: A Systematic Review and Meta-Analysis. Eur. Urol. 77, 403–417. 10.1016/j.eururo.2019.01.049 30773328

[B15] Pfob C. H. Ziegler S. Graner F. P. Köhner M. Schachoff S. Blechert B. (2016). Biodistribution and Radiation Dosimetry of 68Ga-PSMA HBED CC—a PSMA Specific Probe for PET Imaging of Prostate Cancer. Eur. J. Nucl. Med. Mol. Imaging. 43, 1962–1970. 10.1007/s00259-016-3424-3 27207281

[B16] Rahbar K. Ahmadzadehfar H. Kratochwil C. Haberkorn U. Schäfers M. Essler M. (2017). German Multicenter Study Investigating 177Lu-PSMA-617 Radioligand Therapy in Advanced Prostate Cancer Patients. J. Nucl. Med. 58, 85–90. 10.2967/jnumed.116.183194 27765862

[B17] Rasul S. Hacker M. Kretschmer-Chott E. Leisser A. Grubmüller B. Kramer G. (2020). Clinical Outcome of Standardized 177Lu-PSMA-617 Therapy in Metastatic Prostate Cancer Patients Receiving 7400 MBq Every 4 Weeks. Eur. J. Nucl. Med. Mol. Imaging. 47, 713–720. 10.1007/s00259-019-04584-1 31781834 PMC7005080

[B18] Sachpekidis C. Kopka K. Eder M. Hadaschik B. A. Freitag M. T. Pan L. (2016). 68Ga-PSMA-11 Dynamic PET/CT Imaging in Primary Prostate Cancer. Clin. Nucl. Med. 41, e473–e479. 10.1097/RLU.0000000000001349 27607173

[B19] Sandgren K. Johansson L. Axelsson J. Jonsson J. Ögren M. Ögren M. (2019). Radiation Dosimetry of [68Ga]PSMA-11 in Low-Risk Prostate Cancer Patients. EJNMMI Phys. 6, 2. 10.1186/s40658-018-0239-2 30631980 PMC6328430

[B20] Sun M. Niaz M. O. Nelson A. Skafida M. Niaz M. J. (2020). Review of 177Lu-PSMA-617 in Patients With Metastatic Castration-Resistant Prostate Cancer. Cureus. 12, e8921. 10.7759/cureus.8921 32760622 PMC7392183

[B21] Violet J. Sandhu S. Iravani A. Ferdinandus J. Thang S.-P. Kong G. (2020). Long-Term Follow-Up and Outcomes of Retreatment in an Expanded 50-Patient Single-Center Phase II Prospective Trial of 177Lu-PSMA-617 Theranostics in Metastatic Castration-Resistant Prostate Cancer. J. Nucl. Med. 61, 857–865. 10.2967/jnumed.119.236414 31732676 PMC7262220

[B22] Vos E. K. Litjens G. J. S. Kobus T. Hambrock T. Kaa C. A. H.-v. d. Barentsz J. O. (2013). Assessment of Prostate Cancer Aggressiveness Using Dynamic Contrast-Enhanced Magnetic Resonance Imaging at 3 T. Eur. Urol. 64, 448–455. 10.1016/j.eururo.2013.05.045 23751135

[B23] Wang B. Gao J. Zhang Q. Fu Y. Liu G. Shi J. (2020). Diagnostic Value of 68Ga-PSMA PET/CT for Detection of Phosphatase and Tensin Homolog Expression in Prostate Cancer: A Pilot Study. J. Nucl. Med. 61, 873–880. 10.2967/jnumed.119.236059 31757845 PMC7262223

[B24] Zamboglou C. Wieser G. Hennies S. Rempel I. Kirste S. Soschynski M. (2016). MRI Versus 68Ga-PSMA PET/CT for Gross Tumour Volume Delineation in Radiation Treatment Planning of Primary Prostate Cancer. Eur. J. Nucl. Med. Mol. Imaging. 43, 889–897. 10.1007/s00259-015-3257-5 26592938

